# Potential of Exopolysaccharide from *Porphyridium marinum* to Contend with Bacterial Proliferation, Biofilm Formation, and Breast Cancer

**DOI:** 10.3390/md19020066

**Published:** 2021-01-27

**Authors:** Nesrine Gargouch, Fatma Elleuch, Ines Karkouch, Olfa Tabbene, Chantal Pichon, Christine Gardarin, Christophe Rihouey, Luc Picton, Slim Abdelkafi, Imen Fendri, Céline Laroche

**Affiliations:** 1 Institut Pascal, CNRS, SIGMA Clermont, Université Clermont Auvergne, F-63000 Clermont-Ferrand, France; nesrinekarkouch@hotmail.fr (N.G.); christine.gardarin@uca.fr (C.G.); 2 Laboratoire de Biotechnologie Végétale Appliquée à l’Amélioration des Cultures, Faculty of Sciences of Sfax, University of Sfax, Sfax 3000, Tunisia; imenfendri@fss.usf.tn; 3 Centre de Biophysique Moléculaire, CNRS-UPR 4301, 45071 Orléans, France; fatmaelleuch@ymail.com (F.E.); chantalpichon@cnrs-orleans.fr (C.P.); 4 Unité de Biotechnologie des Algues, Biological Engineering Department, National School of Engineers of Sfax, University of Sfax, Sfax 3038, Tunisia; slimabdelkafi@enis.tn; 5 Laboratory of Bioactive Substances, Biotechnology Center of Borj-Cedria (CBBC), BP-901, Hammam-Lif 2050, Tunisia; karkouch_ines@yahoo.fr (I.K.); olfa.tabbene@cbbc.rnrt.tn (O.T.); 6 Normandie University, UNIROUEN, INSA Rouen, CNRS, PBS, 76000 Rouen, France; christophe.rihouey@univ-rouen.fr (C.R.); luc.picton@univ-rouen.fr (L.P.)

**Keywords:** *Porphyridium marinum*, exopolysaccharide, high pressure homogenizer, antibacterial activity, anti-biofilm activity, anti-cancer activity

## Abstract

Exopolysaccharide (EPS) from marine microalgae are promising sources of a new generation of drugs. However, lot of them remain to be discovered and tested. In this study, EPS produced by *Porphyridium marinum* and its oligomers prepared by High Pressure Homogenizer have been tested for different biological activities, i.e., antibacterial, anti-fungal and antibiofilm activities on *Candida albicans*, as well as for their effects on the viability of murine breast cancer cells. Results have shown that all EPS samples present some biological activity. For antibacterial and antibiofilm activities, the native EPS exhibited a better efficiency with Minimum Inhibitory Concentration (MIC) from 62.5 µg/mL to 1000 µg/mL depending on the bacterial strain. For *Candida albicans*, the biofilm formation was reduced by about 90% by using only a 31.3 µg/mL concentration. Concerning breast cancer cells, lower molar masses fractions appeared to be more efficient, with a reduction of viability of up to 55%. Finally, analyses of polymers composition and viscosity measurements were conducted on all samples, in order to propose hypotheses involving the activities caused by the intrinsic properties of polymers.

## 1. Introduction

In recent years, several studies have been conducted on bioactive molecules extracted from microalgal strains such as carbohydrate polymers, proteins, lipids and pigments whose biological and physicochemical properties can be used in the food, cosmetics, medical and pharmacological industries [1,2]. Among these microalgae, red microalgae, especially the genus *Porphyridium* and *Rhodella*, have attracted interest for their richness in sulfated exopolysaccharides (EPS). The molar masses of EPS in these genera is in a range of 2–7 × 10^6^ Da [3]. These anionic sulfated PS contain glucuronic acid and several major neutral sugars such as xylose, galactose and glucose [3,4]. Nevertheless, the structures of these exopolymers have not yet been elucidated except for some oligosaccharidic sequences [5]. These polymers have many potential activities, including antiviral, anti-tumor and antioxidant activities. All of these activities were reported to be linked to these polymers’ physicochemical characteristics such as the degree of sulfation, molecular weight and their rheological behavior [2,4]. However, studies on their antibacterial, anti-fungal and antibiofilm activities remain scarce.

The red microalga *Porphyridium marinum* was studied only for its production of exopolysaccharides and the antiparasitic activity these exopolysaccharides cause for honey bee infection through microsporidia *Nosema ceranae* [6,7]. In this study, EPS produced by *P. marinum* and its oligomers prepared by High Pressure Homogenizer (HPH) were evaluated for several biological activities. These activities were (i) their ability to inhibit the multiplication of Gram (+) and (−) bacterial strains, (ii) the multiplication and biofilm formation of *Candida albicans* yeast and (iii) the proliferation of breast cancer cells. The HPH technique consists of continuously forcing a liquid flow at low velocity using a volumetric pump through a restriction between a seat and a valve, the size of which can be imposed in order to control pressure drop. This technique is a green and non-thermal technology, unlike the most widely used namely chemical and thermal (microwave) methods that are limited by their efficiencies and their toxicities by using chemicals products such as trifluoroacetic acid and sulfuric acid. Moreover, HPH is less costly than enzymatic methods which are simple but often limited by the commercial availability of enzymes, their cost and their sensitivity to denaturation. Furthermore, it is a versatile tool that is commonly used in food and pharmaceutical industries for making emulsions, solid dispersions or cell lyses [8]. Finally, analyses of polymers composition and viscosity measurements were conducted on all EPS fractions, in order to match the activities with the intrinsic properties of polymers and to propose hypotheses on the action mechanism.

## 2. Results

### 2.1. Production of Different Molar Masses Exopolysaccharides by HPH

High Pressure Homogenization, which has been shown to be effective in reducing the molar masses of polysaccharides [9,10], has been applied to EPS produced by the red microalga *P. marinum* in order to obtain lower molar masses exopolysaccharides and therefore to decrease their viscosity. EPS obtained from *P. marinum* were submitted to up to five cycles of HPH at a pressure of 2.7 kbar and three exopolysaccharide fractions were recovered, such as untreated EPS (EPS-0C), EPS after two HPH passes (EPS-2C) and EPS after five HPH passes (EPS-5C).

The number and weight average molar masses (respectively Mn and Mw), gyration (Rg) radius, hydrodynamic (Rh) and intrinsic viscosities ([η]) of samples have been determined by Steric Exclusion Chromatography coupled to multi-angle laser light scattering, viscometry and a differential refractive index (SEC/MALLS/Visco/DRI) and are reported in Table 1.

M_w_ and M_n_ of the native exopolysaccharides were about 1400 and 900 kDa, respectively. Nevertheless, due to an important retention of exopolysaccharide during the filtration (on a 0.45 µm filter), these value are characteristic of less than 10% (Table 1) of the initial mass of the sample before filtration. This low amount of analyzed sample is indicative of the large level of uncertainty on the molar mass determination. However, these values of molar masses seem to be congruent with the results previously reported by Soanen et al. [7] for EPS from *P. marinum* (1800 kDa, recovery rate of 30%). This large loss could be explained by the presence of very large aggregated structures, as we observed high values of hydrodynamic and gyration radii (67 and 150 nm, respectively), but also by the fact that the elution was disperse (i.e., elution on a large volume, thus corresponding to variations in Rg and Rh, but with a quite constant molar mass). Such comportment has also been observed by other authors while analyzing molar masses for microalgae EPS. The work of [11] on *Porphyridium* sp. EPS could be cited in this context, who also suggested the presence of aggregates, as well as a more recent study on *Porphyridium cruentum* [12]. Such large aggregates can be the consequence of hydrophobic associations (the presence of esterified sugar or amphiphilic proteins), hydrogen bonds or complexes between polysaccharides and/or proteins. Besides, we have demonstrated in a previous study on *Flintiella sanguinaria* EPS [13] that large aggregates were present. Conformation analysis in dilute solution with a chaotropic salt (KSCN) led to a partial disaggregation of it, suggesting intermolecular interactions by hydrogen or hydrophobic interactions due to the presence of methyl and acetyl groups on the polysaccharide backbone. However, a protease treatment also confirmed that proteins significantly contribute to this associative structure, but mainly by intramolecular interactions. Thus, even if we have not explored this interaction mechanism more in depth in the present study, the presence of such large aggregates is very likely. In contrast to EPS-0C, the filtration losses of the EPS fractions obtained after two or five passes in the HPH are considerably reduced (about 70% of the samples have been analyzed, see Table 1), leading to more significant values of the polymer physicochemical characteristics. This can be attributed to the decrease in molar masses, even if some smaller aggregates can still remain. As expected, the molar masses of the treated EPS were substantially reduced from 1400 to 550 kDa for M_w_ together with the size (Rh from 67 to 21 nm, Rg from 150 to 41 nm) and [η] from about 1500 to 150 mL/g). Therefore, the HPH treatment was clearly effective to reduce both molar masses, hydrodynamic and gyration radius of polymers by a factor of 2.5, 3 and 3.5, respectively. However, the difference between the 2nd and 5th passage appears to not be really significant.

### 2.2. Structural and Physico-Chemical Characterization of EPS Samples

The purity of the native EPS (EPS-0C) was first evaluated, showing that our sample was constituted of 62% sugars. Additionally, 9% ± 0.04 of proteins were found to be present in the polysaccharide extract. This percentage is consistent with those generally found in the literature for EPS from red microalgae [2]. Some authors have even suggested that these proteins could be covalently bound to the polysaccharidic moiety [14]. No further purification was then applied to the sample.

Biological activity of polysaccharides is often attributed to the presence of sulfate groups and uronic acids in their structure. The composition of the 3 samples was analyzed by colorimetric assays, HPAEC-PAD and FTIR. As shown in Table 2, no modification of the global composition was noticed since all samples were composed of around 22% of uronic acids, and 9% of sulfate groups. The HPAEC chromatograms are provided as Supplementary Materials.

Concerning monosaccharides composition, the exopolysaccharides of *P. marinum* consisted mainly of xylose, galactose and glucose as previously reported by Soanen et al. [7]. Only slight differences were noticed between treated and untreated samples, which were considered to not be significant. This result is in accordance with the fact that no additional purification step was included after HPH treatment, so all monosaccharides present in the native sample remained in the treated ones. Differences between uronic acids content obtained by colorimetric assay (22% in weight) and HPAEC (~5% molar ratio glucuronic acid) could be attributed to the presence of an unidentified peak in HPAEC corresponding to another acidic monosaccharide. This hypothesis is supported by the fact that methylated glucuronic acid (2-*O*-Me-GlcA) has been described for *Porphyridium cruentum* [15], and more recently, [16] have observed the presence of a methylated uronic acid in the EPS from *Porphyridium sordidum*. Moreover, this peak has been already observed by authors while analyzing the EPS from *Flintiella sanguinaria* (another red marine microalgae). During this study, it was concluded that this peak should correspond to a methylated and/or acetylated glucuronic acid. This hypothesis was formulated because the analysis of the desubstituted sample (specific treatment to release methyl and acetyl groups for their quantification), led to the disappearance of this peak and increase in the area of glucuronic acid peak. However, the lack of commercial standards prevented its formal identification and quantification [13].

**Figure 1:**
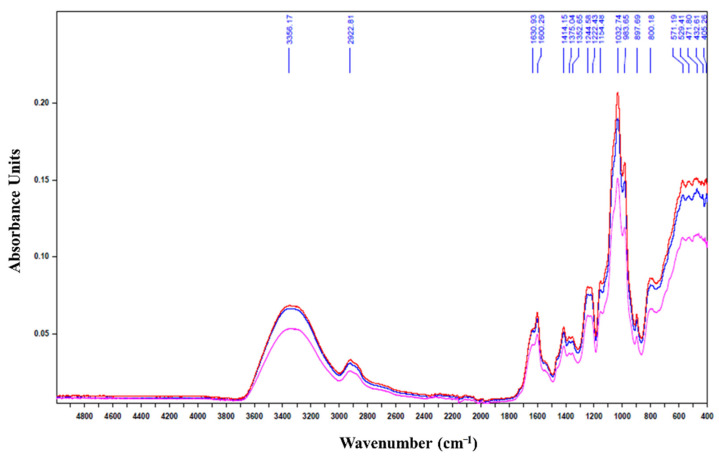
Infrared spectrum of exopolysaccharide (EPS) before and after passage in the high pressure homogenizer. Blue: EPS-0C, Red: EPS-2C, Pink: EPS-5C.

Finally, infrared spectrum analysis indicated that the HPH-depolymerized products had the same footprint as the native exopolysaccharide (Figure 1).

No difference was noticed in the absorption bands detected between 1630 and 1414 cm^−1^ which are characteristic of the asymmetric vibrations of the protonated and deprotonated carboxylic groups linked to the presence of uronic acids [17,18]. The 1222.43 cm^−1^ band characteristic of the S=O sulfate groups [19] of the red microalgae EPS was retained for the two treated polysaccharides: EPS-2C and EPS-5C. Also, the absorption bands detected at 897.69 cm^−1^, characteristic of the deformation of the β-C_1_ anomeric bonds [20,21], were well preserved during the depolymerization. The bands observed at 1154 cm^−1^ are characteristic of the glycosidic structure (C-O-C). In fact, the presence of these bands despite the depolymerization by HPH shows that only certain glycosidic bonds have been affected and altered. These results confirm the still high molar masses of EPS-2C and EPS-5C determined by SEC/MALLS (Table 1). These findings are consistent with those observed by [22] while extracting fucoidans from the seaweed *Nemacystus decipients* by several cycles of HPH.

**Figure 2:**
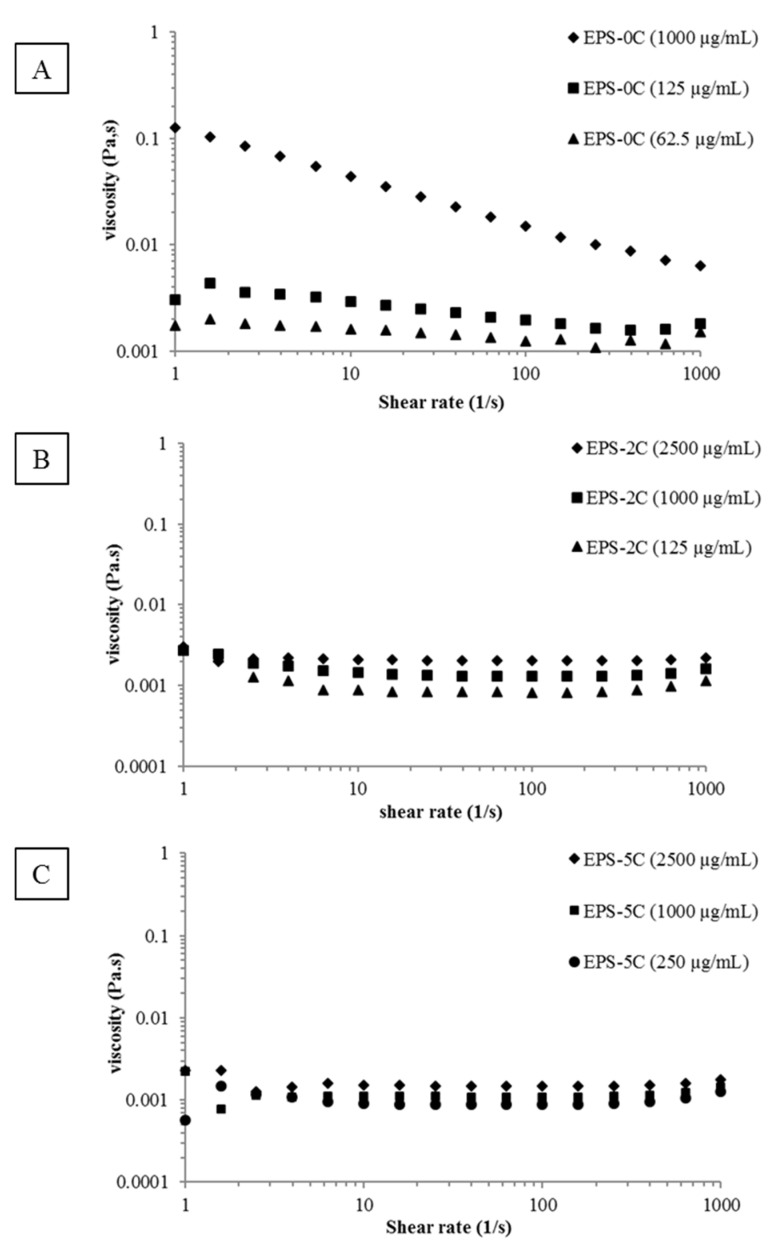
Shear flow behavior of EPS samples as a function of concentrations. (**A**): Native EPS (EPS-0C) at 1000 µg/mL (♦), 125 µg/mL (■) and 62.5 µg/mL (▲); (**B**): EPS-2C at 2500 µg/mL (♦), 1000 µg/mL (■) and 125 µg/mL (▲); (**C**): EPS-5C at 2500 µg/mL (♦), 1000 µg/mL (■) and 250 µg/mL (●).

Apart from functional groups, the viscosity of polymers has been suggested to play a role in some biological activities. As an example, it has been demonstrated by Sun et al. [23] that by decreasing the average molar masses of the polymer, the viscosity was considerably reduced (in agreement with our results, see Table 1) and consequently the antioxidant activity gradually improved. Thus, the rheological behavior of the various exopolysaccharide extracts (EPS-0C, EPS-2C and EPS-5C) was explored in flow mode by plotting the viscosity curves as a function of the shear rate (Figure 2). The different samples were prepared at the same concentration (1 mg/mL) for a comparative study but also at some active concentrations that will be highlighted later in this paper (125 and 62.5 µg/mL for EPS-0C, 125 and 2500 µg/mL for EPS-2C as well as 250 and 2500 µg/mL for EPS-5C).

As shown in Figure 2A, the EPS-0C at the highest concentration, (i.e., 1000 µg/mL) evidences a non-Newtonian and shear-thinning behavior that means the decrease of the viscosity of the solution with the increase of the shear rate. Moreover, the absence of a Newtonian plateau at low shear rates could be indicative of a yield stress behavior in agreement with a connected structure of the fluid. This result could be correlated to the high aggregation tendency showed in a diluted regime (SEC/MALLS/Visco/DRI measurements). Some papers described rheological behavior of microalgae EPS, including from *Porphyridium* species (*P. cruentum* [12], *P. sordidum* and *P. purpureum* [24]), but all were conducted at greater concentrations than in the present study (0.125% or more), leading to difficulties in comparing viscosity values. However, they also observed a shear thinning behavior [12,23,25,26]. At lower concentrations (for 125 µg/mL and 62.5 µg/mL), EPS-0C presents a quite Newtonian profile. Thus, the viscosity was found to be considerably decreased from 44.22 mPa.s (1000 µg/mL) to 2.95 mPa.s (125 µg/mL) and 1.62 mPa.s (62.5 µg/mL) at a shear rate of 10 s^−1^ (Figure 2A). This result is in agreement with Balti et al.’s [27] study, who observed a decrease in viscosity from 18.7 to 4.6 mPa.s when the sugar concentrations decreased from 1.74 to 0.48 g/L at a shear rate equal to 15 s^−1^. On the other hand, the viscosities of EPS-2C and EPS-5C solutions have shown a linear relationship with the shear rate typical of Newtonian fluids (Figure 2B,C). Thus, decreasing the molar masses of EPS by HPH led to a significant impact on viscosity which decreased from 44.22 mPa.s for EPS-0C to 1.12 mPa.s (just slightly higher than water viscosity) for EPS-5C for a shear rate equal to 10 s^−1^. As for molar masses determination, no more effect between two and five passages has been detected with this rheological study. Partial depolymerization of EPS from *Porphyridium* sp. has been shown to strongly affect viscosity, with, for the lower molecular weight samples, a comportment typical of Newtonian fluids [23]. Villay et al. [8] showed the same effect on other types of polysaccharides (guar gum, hydroxyethylcellulose (HEC), sodium carboxy-methylcellulose (Na-CMC), sodium alginate (Na-alginate) and gum Arabic) treated with HPH. The effect of number of treatment cycles was similar for all the polysaccharides. The first treatment always had the strongest impact on viscosity reduction. Then, no effect on the zero-shear viscosity “*η*_s_” was observed after 2 and 3 cycles. Finally, a slight decrease of *η*_s_ was observed between cycles 4 and 6. At last, only a slight difference in viscosities of the treated samples could be detected as a function of concentration according to the expected dilute regime of concentration. These results were in accordance with the intrinsic viscosity (η) measurements (Table 1) which decreased significantly after depolymerization from 1480 to 155 mL/g (Table 1).

### 2.3. Biological Activities

#### 2.3.1. Antibacterial and Anti-*Candida Albicans* Activities

The three EPS fractions were screened for their ability to inhibit the growth of four bacterial strains (*Staphylococcus aureus* ATCC 29213, methicillin resistant *Staphylococcus aureus* (SMR), *Escherichia coli* ATCC 25922 and *Salmonella* Enteritidis ATCC 13076) and the yeast *Candida albicans* ATCC 10231.

All 3 EPS samples showed an antibacterial activity against the different bacterial strains tested. However, no antifungal activity against *Candida albicans* was detected. The most active was the native EPS that exhibited antibacterial activity against *E. coli* and *Salmonella* (Gram (−)) at MIC (Minimum Inhibitory Concentration) of 62.5 µg/mL and 125 µg/mL, respectively. This extract has also shown growth inhibition of Gram (+) bacteria at MIC 125 µg/mL for *S. aureus* and 1000 µg/mL for SMR. EPS-2C inhibited Gram (−) bacteria at a concentration of 2500 µg/mL and exhibited activity against *S. aureus* at a MIC of 1250 µg/mL. Finally, EPS-5C showed an antibacterial activity against *E. coli* and *S. aureus* at a concentration of 2500 µg/mL (Table 3). The MIC value of the different EPS samples was higher than that of the reference antibiotics Cefazolin and Amphotericin B that were used as positive controls. Even if the antimicrobial potential of these conventional antimicrobial agents was higher than that of EPS, the later could represent a natural and safer molecule without secondary effects.

**Figure 3:**
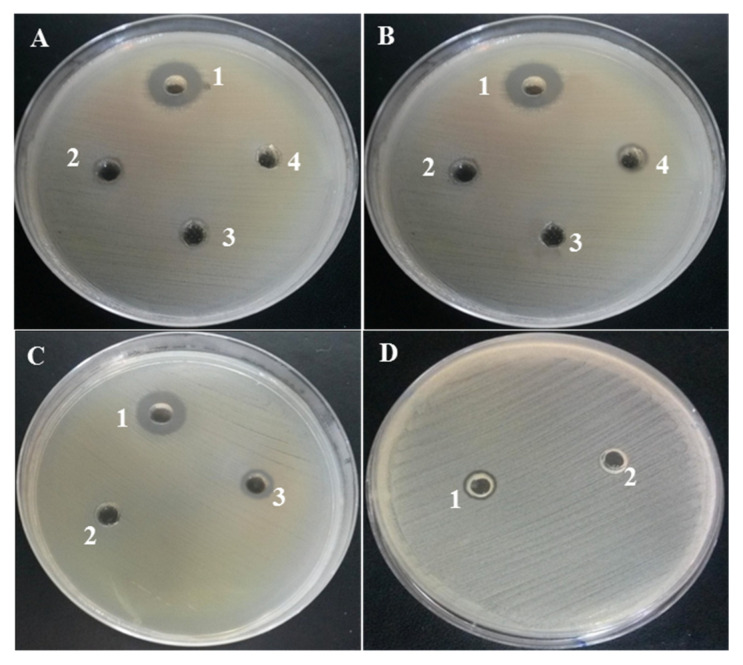
Zones of inhibition of the different bacterial strains affected by the exopolysaccharide extracts. (**A**): Activity against *E. coli*, 1: EPS-0C, 2: EPS-2C, 3: EPS-5C, 4: water, (**B**): Activity against *S. aureus*, 1: EPS-0C, 2: EPS -5C, 3: water, 4: EPS-2C, (**C**): Activity against *Salmonella*, 1: EPS-0C, 2: water, 3: EPS-2C, (**D**): Activity against SMR, 1: EPS-0C, 2: water.

These antibacterial activities were also demonstrated by the appearance of an inhibition zone on agar medium using the well diffusion method at MIC concentrations obtained previously (Figure 3). According to this test, it clearly appeared that the various exopolysaccharide fractions have an inhibitory effect on the growth of the bacteria, manifested by the formation of an inhibition zone around the wells. This area depended on the sensitivity of the bacterial strain to the EPS extract and its concentration. Indeed, *Escherichia coli* ATCC 25922 was found to be the most sensitive Gram (−) bacterium to EPS-0C with a MIC of 62.5 µg/mL while *Staphylococcus aureus* ATCC 29213 was the most sensitive Gram (+) bacterium to EPS-0C with a MIC equal to 125 µg/mL. This sensitivity was revealed by an inhibition zone which was the most extensive in comparison with those of the other polysaccharide extracts (Figure 3), which were active only at concentrations greater than or equal to 1000 µg/mL. Thus, the native EPS (EPS-0C) seemed to be the most active compared to other exopolysaccharide extracts (EPS-2C and EPS-5C).

#### 2.3.2. Inhibition of Biofilm Formation by *Candida Albicans* ATCC 10231

**Figure 4:**
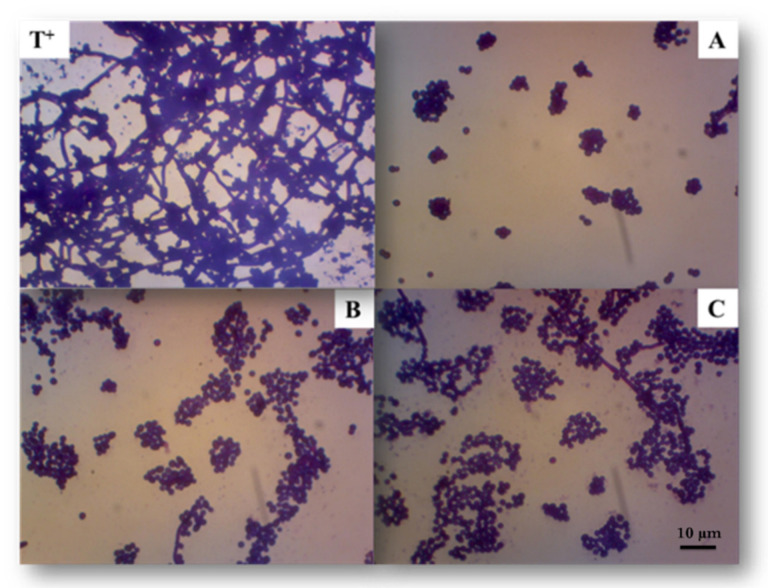
Representative reverse microscopy images of the biofilm formed by *C. albicans* ATCC 10231 in absence (positive control: T^+^) or in the presence of exopolysaccharides at active concentrations. (**A**): EPS-0C at 31.3 μg/mL; (**B**): EPS-2C at 125 μg/mL and (**C**): EPS-5C at 250 μg/mL. The assay was performed on a 96-well plate for 24 h at 37 °C and the adherent biofilm cells were stained by crystal-violet.

*P. marinum* EPS extracts were tested for their ability to inhibit *Candida albicans* biofilm formation by the 96-well plate crystal violet test. The three fractions (EPS-0C, EPS-2C and EPS-5C) were revealed to be active against the formation of *Candida albicans* biofilm (Figure 4) without affecting the planktonic growth of this yeast, as no mortality had been observed previously (Table 3).

**Figure 5:**
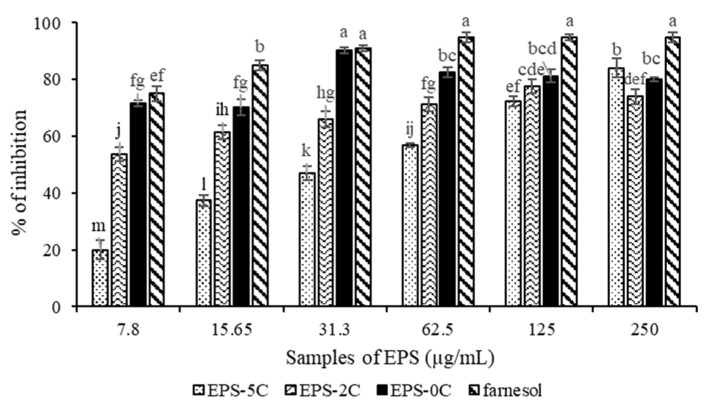
Percentage of inhibition of *Candida albicans* biofilm formation by different molar masses EPS from *P. marinum* (EPS-0C, EPS-2C and EPS-5C) and at different concentrations between 7.8 and 250 µg/mL. Farnesol was used as a positive control. Results were expressed as mean ± standard deviations of triplicate determinations. Different lowercase letters stand for significantly different values (Duncan multiple range test, *p* <0.05).

Based on this observation, active EPS do not appear to target cell viability; they instead represent compounds whose mechanism of action would be the inhibition of the virulent form of *C. albicans*, in particular the transition from the yeast form to the mycelial form. As shown on Figure 5, the native polymer EPS-0C was the most active compared to other fractions (EPS-2C and EPS-5C). In fact, it has shown an inhibition of 90 ± 1% at a concentration equal to 31.3 μg/mL, not significantly different from the efficiency of farnesol, used as a positive control, which inhibits 91% of *C. albicans* biofilm formation at 31.3 µg/mL concentration. However, EPS-2C inhibited the formation of biofilm by up to 78 ± 2% at 125 μg/mL and EPS-5C had an activity of 83 ± 3% at a concentration of 250 μg/mL.

#### 2.3.3. T1 Mammary Carcinoma Cells Sensitivity towards EPS Samples

**Figure 6:**
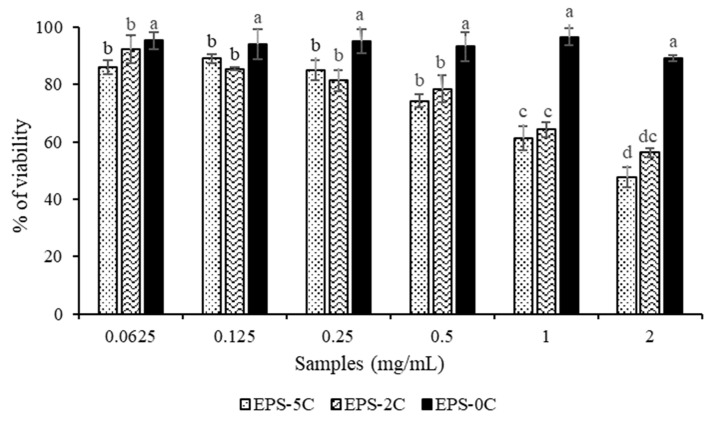
Percentage of viability of murine mammary carcinoma cells 4T1 under the action of EPS samples from *P. marinum* (EPS-0C, EPS-2C and EPS-5C), at concentrations between 0.0625 and 2 mg/mL. Results were expressed as mean ± standard deviations of triplicate determinations. Different lowercase letters stand for significantly different values (Duncan multiple range test, *p* < 0.05).

The results of the in vitro test of the different EPS fractions on a murine breast carcinoma 4T1 cell lines have shown an antiproliferative activity on cancer cells mainly for EPS-2C and EPS-5C fractions (Figure 6). Indeed, the EPS-0C extract at the concentration of 2 mg/mL showed no significant inhibition of cancer cells with a percentage of viability equal to 85 ± 0.8%, which was found to not be significantly different from the viability obtained at the lower concentration (>90%). This result highlights the fact that the native EPS has no activity on carcinoma cells proliferation. In contrast, the antiproliferative activity was improved with the EPS-2C and EPS-5C exopolysaccharides having lower molar masses, as they were found to reduce the cell viability by 51 ± 0.75% and 45 ± 0.82%, respectively at a concentration of 2 mg/mL. As shown on Figure 6, EPS-2C and EPS-5C inhibited the proliferation of cancer cells in a concentration-dependent manner. In fact, the more the concentration (from 0.0625 to 2 mg/mL) increased, the more the antiproliferative activity of cancer cells increased, but with no statistically different behavior between the 2 samples.

Cytotoxicity of EPS samples was assayed on mammalian cell line Vero. Viability of cells was evaluated for each sample and at different concentrations (up to 5 mg/mL). Results showed that on the whole range of concentrations tested (between 9.75 to 5000 µg/mL), and for the 3 extracts (EPS-0C, EPS-2C and EPS-5C), no significant difference in viability was observed, and that it was almost 100% in all cases. As the CC50 (The 50% cytotoxic concentration, defined as the sample concentration able to reduce the cell viability by 50% when compared to an untreated control) of the samples were evaluated to be >5 mg/mL, and that no cytotoxic effect was observed for these samples in the range of concentrations used for the XTT test, the decrease in cell viability of carcinoma cells (about 50%) observed for 2 mg/mL concentrations could not be attributed to a cytotoxicity of the extracts and thus confirmed the antiproliferative effect.

## 3. Discussion

EPS from red microalgae showed several biological activities [28,29,30,31,32,33] that were generally linked to their molecular weight, rheological behavior [23,34] and their content in sulfate groups and uronic acids. In our study, we evaluated the global and monosaccharide composition, the FTIR footprint, the amount of uronic acids and sulfate groups and the molecular masses of our samples. These results are in accordance with other studies on *P. marinum* EPS [6,7]. EPS from microalgae are complex molecules (much more complex than polysaccharides from other sources), making their complete characterization really challenging. Only a few numbers of EPS coming from microalgae have been studied for glycosidic linkages and none of these studies have led to a complete and defined structure [2]. The cause of this poor knowledge of EPS structures is linked to the fact that these heteropolymers often contains 5 to 10 different monosaccharides, numerous non-sugar substituents such as sulfate, methyl, acetyl and/or pyruvyl groups, and they apparently lack a repeating unit. In a drug development approach, it is mandatory to have fully characterized molecules at the molecular level, including resolution of the glycosidic linkages. However, methods that are classically applied successfully to polysaccharides from other sources (NMR, MALDI-TOF, GC-MS/EI) actually fail to resolve the structure of EPS from microalgae [2]. Even if the elucidation of this complete structure is still lacking, we intended to highlight a potential relationship between structure, physico-chemical properties and activities.

Many researchers have reported that minor changes in molar masses, or viscosity can have positive effects on antitumor activity. In this study, we demonstrate that EPS-2C and EPS-5C show an anti-proliferative activity against breast cancer cells at a high concentration of 2 mg/mL (Figure 6). This activity could be attributed to their lower molar masses and therefore to their low viscosity, thereby increasing their absorption and permeation ability. By further decreasing the molar masses of EPS from *P. marinum*, it could then be expected to increase this anti-proliferative effect. In 2009, Gardeva et al. [34] reported the anti-tumor activity of *Porphyridum cruentum* exopolysaccharide. They discovered that this sulfated polymer strongly inhibited proliferation of Graffi myeloid tumors in vitro and in vivo. These authors hypothesized that the anticancer activity of the exopolysaccharide extracted from *Porphyridium cruentum* could be associated with its immunostimulatory action as well as its direct cytotoxic properties. In 2012 [35], the anti-tumor and immunomodulatory activities of *P. cruentum* exopolysaccharides of different molar masses were evaluated on the mouse model carrying the S180 tumor in vivo and on the activation of peritoneal macrophages in vitro. The degraded EPSs showed a net immunomodulation dependent on their molar masses as well as the administered concentration. The smallest fragment of 6.53 kDa had the strongest immuno-reinforcing activity, which was in agreement with the results obtained in our study. Other studies have confirmed that many types of polysaccharides, such as lentinan, *Spirulina maxima* polysaccharide and *Chondrus ocellatus* λ-carrageenan, exhibited significant antitumor activity for low molar masses samples [36,37,38]. In general, the antitumor effects of polysaccharides involve several mechanisms such as modification of the biochemical character of the cell membrane, induction of differentiation and apoptosis of tumor cells and regulation of cell signaling pathways. Nevertheless, immunomodulation is generally considered to be the most important mechanism [39]. It will then be interesting to test lower molar masses samples for antitumoral activity, and to evaluate the immunomodulatory effect to go further in the comprehension of the mechanism.

Bacteria are very common microorganisms in the pathogenic state. The best known and encountered bacteria in the medical sector is *Staphylococcus aureus* which is involved in nosocomial infections [40], but which can also be responsible for food poisoning just like *Escherichia coli* and *Salmonella* [41,42]. However, some bacteria also have developed an increased resistance to clinical antibiotics like methicillin, rendering it ineffective [43]. *Candida albicans* is the most common microorganism among fungal infections, causing high mortality and morbidity especially in immunocompromised patients [44]. The formation of the biofilm represents the virulent form of the pathogen contributing to the pathogenesis of candidiasis. The formation of the *Candida albicans* biofilm is favored by the passage from the unicellular yeast form (blastopore) to the mycelial form (hyphe and pseudohyphe) [45,46], with the ability to strongly adhere to biological surfaces or inert surfaces of medical devices [47]. Biofilm formation leads to high levels of resistance to most conventional antifungal agents, mainly fluconazole and amphotericin B, which have several limitations in terms of efficacy, toxicity, drug interaction and high cost. Thus, the search for alternative strategies and the development of new and more effective antifungal and antibacterial agents is necessary for better therapeutic management. For these reasons, EPS fractions from *P. marinum* were also tested for their antimicrobial and antibiofilm activities. It was found that the native EPS as well as the depolymerized EPS (EPS-2C and EPS-5C) provided an antibacterial and antibiofilm activity with different efficiency levels (Table 3, Figure 5). Our EPS can thus be considered as a powerful inhibitor of bacterial multiplication compared to other exopolysaccharides extracted from other microalgae. De Jesus Raposo et al. [48] indicated that the EPS produced by *Porphyridium cruentum* exhibited antibacterial activity against Gram (+) (*S. aureus*) and Gram (−) bacteria (*E. coli* and *S.* Enteritidis). Indeed at 1% (*w*/*v*), it clearly inhibited *S.* Enteritidis, reducing CFU to 19%. However, the concentration used in the present study was much lower. The most active fraction was the native EPS at low concentrations that were not showing high viscosity (31.3 µg/mL for the inhibition of the biofilm of *Candida albicans* 110231, 62.5 µg/mL for *anti-Escherichia coli* ATCC 25922 multiplication and 125 µg/mL for anti-*Salmonella* Enteritidis ATCC 13076 and anti-*Staphylococcus aureus* ATCC 29213 growth). Very few polysaccharides from microalgae were described as negatively impacting biofilm formation. The only available study has shown that the cell-bound polysaccharide from *Navicula phyllepta* specifically inhibited biofilm formation from the bacteria *Flavobacterium* sp., while it stimulated biofilm development by *Roseobacter*, and *Shewanella* genera [49]. In comparison with the literature, the efficiency of EPS-0C to inhibit biofilm formation by *C. albicans* observed in this study was greater (>90%), and this for much lower applied doses (31.3 μg/mL). However, the active dose of EPS-2C and 5C was found to be greater than for EPS-0C, showing that decreasing the molar mass has a negative impact on their efficiency.

The native EPS was also active against *Staphylococcus aureus* methicillin resistant at a concentration equal to 1000 µg/mL (Table 3) showing a significant viscosity (Figure 2A). Therefore, in addition to the functional groups (sulfate and uronic acids, see Table 2) present on EPS-0C, the role of viscosity cannot be excluded from its inhibiting properties on the multiplication of SMR. The rheological behavior of the EPS-2C and EPS-5C fractions was always the same regardless of the applied concentration (2500 µg/mL, 1000 µg/mL or 125 µg/mL) (Figure 2B,C). At these concentrations, these samples have a low viscosity and exhibit a Newtonian behavior. Therefore, the biological activity of these EPSs against bacterial strains and the inhibition of *Candida albicans* biofilm formation could be attributed to their chemical composition, probably primarily to the sulfate and uronic acids groups, as several studies have relied on biological activity with the presence of sulfate groups [33,48,50,51]. Nevertheless, the sulfation degree may not be the only parameter to be considered to explain the biological activity of polysaccharides. Their position and therefore their “accessibility” can improve or not improve the biological activity of the EPS, as demonstrated by [6]. Moreover, several different mechanisms may exist, as non-sulfated polysaccharides (such as chitosan) have exhibited significant efficiency as an antibacterial and antibiofilm agent. For example, Costa et al. [52] have shown that the highest percentage inhibition of biofilm formation by *C. albicans* (66.94%) was obtained for high molar masses chitosan at 0.5 mg/mL and the lowest inhibition percentage (37.97%) was obtained for the low molar masses at 0.75 mg/mL, which was in agreement with our results. Cobrado et al. [53] also showed that low molar masses chitosan (107 kDa) was able to reduce *C. albicans* biofilm formation up to 41% at a 2.5 mg/mL concentration. For antibacterial activities, results are sometimes controversial, with depending on studies, greater efficiency on Gram (+) bacteria [54,55] or on Gram (−) ones [56,57]. In fact, the antimicrobial activities of chitosan would be greatly dependent on its physical characteristics, most notably weight average molar masses (Mw) and degree of deacetylation (DD) (for a review, see [58]). Therefore, the antimicrobial mechanisms and structure-function relationships of polysaccharides remain to be elucidated.

## 4. Materials and Methods

### 4.1. Extraction of Exopolysaccharides

The exopolysaccharide was recovered from a culture of red microalga *Porphyridium marinum*, obtained from Culture Collection of Algae and Protozoa (http://www.ccap.ac.uk/). Cultivation was conducted in 2 L Erlenmeyer flask, containing 1 L of Pm medium [7], under continuous light at 150 µmole photons/m²/s, stirring at 110 rpm and at a temperature of 20 °C. The medium was inoculated with 100 mL of a subculture. As previously described [7], synthesis and accumulation of EPS occurred after nitrogen deprivation and entry in a stationary phase. Culture was stopped after 30 days, centrifugated (10,000× *g*, 30 min, 20 °C), and the supernatant was desalted and concentrated using a tangential filtration cassette (Vivaflow 200, Sartorius, Göttingen, Germany) having a cutoff threshold of 100 kDa [7]. Finally, the resulting EPS solution was lyophilized before storage at room temperature.

### 4.2. Preparation of Different-MW Exopolysaccharides

The exopolysaccharide of *P. marinum* was treated using a High Pressure Homogenizer (HPH, 2.7 kbars, TS HAIVA, Constant Systems LTD, Daventry, UK) to obtain polysaccharide fractions of different molar masses. A polysaccharide stock solution was prepared at a concentration of 2 g/L. Up to five successive cycles were used to obtain 3 samples which were subsequently recovered and lyophilized. The corresponding degraded samples are labeled as follows, according to the number of cycles in the HPH:

- EPS-0C for the undegraded sample,
- EPS-2C for the polysaccharide after two cycles,
- EPS-5C for the polysaccharide after five cycles.

### 4.3. Exopolysaccharides Characterization

#### 4.3.1. Total Sugars, Neutral Sugars, Uronic Acids, Sulfate and Proteins Content

Total sugar content of samples was evaluated by the resorcinol method using glucose as standard [59]. Uronic acids and neutral sugar contents of EPS extracts were assayed with meta-hydroxyldiphenyl (m-HDP) and resorcinol as described by, respectively, [60] and [59] using glucose and glucuronic acid as standards. In these assays, glycosidic linkages are broken during high temperature sulfuric acid treatment and dehydration of sugar units leads to furfural compounds formation. These latter interact by condensation with phenolic compounds (resorcinol or m-HDP depending on the assay), giving colored compounds that can be measured respectively at 450 or 520 nm. Briefly, for the m-HDP assay, 200 µL of sample are mixed with 1 mL of a 0.12 M borax (Na_2_B_4_O_7_,10H_2_O) prepared in 96% (*w*/*v*) sulfuric acid. After a 1 h incubation at 90 °C, 200 µL of m-HDP solution are added and absorbance measured at 520 nm. The m-HDP solution is prepared just before assay by diluting 102 µL of m-HDP stock solution (100 mg of m-HDP solubilized in 1 mL DMSO, kept at 4 °C) with 5 mL of 80% (*w*/*v*) sulfuric acid. The results were expressed in mg/g of d-glucuronic acid equivalent (GlcAEq). For resorcinol assay, 200 µL of sample are mixed with 200 µL of a 6 g/L resorcinol solution and 1 mL of 80% (*w*/*v*) sulfuric acid. After 30 min at 90 °C, followed by 30 min at room temperature, 1.4 mL of water are added and absorbance at 450 nm is measured. The results directly expressed in mg/g of D-glucose equivalent (GlcEq) correspond to the total sugars amount. As the uronic acids interfere with the assay, a glucuronic acid standard curve was performed following the same procedure, and the corrective formula described by [61] was applied, in order to access the neutral sugars amount.

Sulfur content was determined by the turbidimetric method [62] using K_2_SO_4_ as a standard. Results were expressed in mg/g of SO_4_ equivalent. Briefly, 120 mg of samples were hydrolyzed with 3 mL of 2 M chlorhydric acid for 2 h at 100 °C. After centrifugation (10,000× *g*, 30 min), 1 mL of supernatant is added to 9 mL of milliQ water, 1 mL of 0.5 M HCl, and 0.5 mL of BaCl_2_/gelatin reagent (150 mg of gelatin dissolved in 50 mL of water at 70 °C, kept 16 h at 4 °C, and finally mixed with 0.5 g of BaCl_2_). After 30 min incubation at room temperature, sulfate groups released during the hydrolysis of polysaccharides form a precipitate of barium sulfate, which can be detected at 550 nm. Protein content was evaluated by the Lowry method [63] using bovine serum albumin (BSA) as a standard.

#### 4.3.2. Monosaccharide Composition of EPS

The identification and quantification of the constitutive monosaccharides of *P. marinum* EPS was conducted by high performance anion exchange chromatography (HPAEC) on an ICS 3000 (Dionex, Sunnyvale, CA, USA). Exopolysaccharides were hydrolyzed with 2 M TFA at 120 °C for 1 h 30 min, neutralized with 35% ammonia and then centrifuged at 14,000× *g* for 15 min. The recovered supernatant was filtered at 0.22 μm before injection. The elution was carried out with a pre-column and a CarboPac PA1 column (4 × 50 mm and 4 × 250 mm, respectively) whose stationary phase is an anion exchange resin. Samples were eluted isocratically with 18 mM NaOH for 25 min, followed by a linear gradient between 0 and 0.5 M sodium acetate in 200 mM NaOH for 20 min to elute acidic monosaccharides. Run was followed by 15 min washing with 200 mM NaOH. The eluent flow rate was kept constant at 1 mL/min. A range of external standards (Fucose, Arabinose, Galactose, Glucose, Rhamnose, Xylose, Mannose, Fructose, Ribose, Galactosamine, Glucosamine, N-AcetylGlucosamine, N-AcetylGalactosamine, Galacturonic acid and Glucuronic acid) with concentrations ranging from 0.001 to 0.01g/L, and then the addition of internal standards in the samples enabled qualitative and quantitative analysis of the composition of polysaccharides.

#### 4.3.3. Molar Masses Determination

Molar masses and polydispersity index of EPS were analyzed by a size exclusion chromatography coupled to a multiangle laser light scattering detector, a viscometric detector and a differential refractive index detector (SEC/MALLS/Visco/DRI). The polysaccharide solutions were prepared at 0.5 mg/mL (dilute regime) in the chromatographic eluent (i.e., LiNO_3_ at 0.1 M, filtered on 0.1 µm) under soft magnetic agitation at 60 °C during 4 h and then filtrated on a 0.45 µm filter unit (regenerated cellulose). An automatic sample injector (SIL 20A, Shimadzu, Kyoto, Japan) equipped with a 500 μL injection loop was used for injection. The solvent was driven by a pump (LC 10Ai, Shimadzu, Kyoto, Japan) at a flow rate of 0.5 mL/min. The sample was first drawn into a pre-column (Shodex OHPAK SB-G) before being directed to two standard steric exclusion columns (Shodex OHPAK SB 804 and 806 HQ). At the outlet of the column, the compounds were quantified by a differential refractometer (DRI 10A, Shimadzu, Kyoto, Japan), a multi-angle laser light scattering detector (Dawn^®^ heleos, Wyatt technology Corp., Santa Barbara, CA, USA) equipped with a K5 cell of 50 μL, a red source (Ga-As 690 nm) of 5 mW, 18 measurement diodes and a viscosity detector (Viscostar 2, Wyatt Technology Corp., Goleta, CA, USA). The data obtained was processed with the Astra 6.1.1 software which provided the weight and number average molar masses (M_n_ and M_w_, respectively) and also the gyration (Rg) radius and both intrinsic viscosities ([η]) and hydrodynamic (Rh) based on the viscosity detector.

#### 4.3.4. Infrared Spectrometry Footprint (FTIR)

Infrared measurements were made using a VERTEX 70 (Bruker, Billerica, MA, USA) spectrometer. The samples were analyzed on an ATR A225 diamond module. IR spectra (50 scans) were obtained at room temperature (referenced against air) over the 500–4000 cm^−1^ range. The spectra were analyzed with the OPUS 7.2 software.

#### 4.3.5. Shear Flow Behavior of EPS Samples

The shear flow behavior of samples (viscosity measurements as function of shear rate) was determined using an AR-G2 rheometer (TA Instruments Ltd., Hertfordshire, UK). Measurements were conducted using a steel cone-plate (40 mm radius, 2°, gap 50 µm) geometry. The temperature was set at 30 °C, with a circulating bath or a Peltier system depending on geometry. The viscosity measurements were made over a shear rate range of 1 to 10^3^ s^−1^.

### 4.4. Biological Activities

#### 4.4.1. Antibacterial Activity

Four bacterial strains: *Staphylococcus aureus* ATCC 29213, methicillin-resistant *Staphylococcus aureus* (SMR), *Escherichia coli* ATCC 25922 and *Salmonella* Enteritidis ATCC 13076, were used as indicator strains for the determination of the antibacterial activity. Exopolysaccharides samples were tested for antibacterial activity by using the well diffusion method and the minimum inhibitory concentration (MIC) using the microdilution method on sterile 96-well plate [64]. Briefly, on Petri dishes containing solid Luria broth (LB) medium previously inoculated with the bacterial inoculum, 6 mm wells were dug in the medium and then sealed with a thin layer of liquefied soft agar to limit the diffusion of the sample under the solid medium. Samples with 50 µL of exopolysaccharides were placed in each well. Sterile distilled water was used as a negative control. The diameters of the inhibition zones were measured after 18 h of incubation at 30 °C.

To determine the MIC, bacterial strains were grown in LB medium at 30 °C. 10^5^ CFU/mL of each bacterial suspension were incubated for 24 h at 30 °C in the presence of the test sample at concentrations ranging from 7.81 to 1000 μg/mL. Wells containing the bacterial strain alone were used as a positive control, while wells containing only the culture medium were considered as a negative control. MIC values were determined for the lowest concentration of the extract, showing complete inhibition of bacterial cell growth.

#### 4.4.2. Anti-Candida Albicans Activity

Detection of antifungal activity against *Candida albicans* 10231 was performed using the microdilution method on a 96-well plate, as previously described by Grieco et al. [65]. Briefly, a culture of *C. albicans* (30 °C in WB medium) is diluted in the same growth medium to reach 10^5^ UFC/mL and the suspension is introduced in each well, with different concentrations of samples between 7.81 and 1000 µg/mL and the plate is incubated 24 h at 37 °C. Two controls were included. The negative control corresponds to the growth medium, and the positive control contains *C. albicans* in the growth medium. MIC values were determined as the lowest concentration of the extract showing complete inhibition of *Candida* cell growth.

#### 4.4.3. Inhibition of Biofilm Formation

Biofilm quantification was performed using the crystal violet staining assay on a 96-well polystyrene plate, as demonstrated by Jin et al. [66]. Indeed, a suspension of *Candida albicans* with a final concentration of 10^5^ CFU/mL was prepared in the RPMI 1640 culture medium (R8758, Sigma-Aldrich, St. Quentin Fallavier Cedex, France). Fifty microliters of *C. albicans* inoculum was distributed into each well of the microplate in the presence of different exopolysaccharides samples, with concentrations ranging from 7.81 to 1000 μg/mL to have a final volume of 100 μL. The plate was then incubated at 37 °C for 24 h. After incubation, crystal violet staining was performed to demonstrate the inhibitory effect of biofilm formation. Briefly, the culture medium was removed and each well was washed twice with PBS buffer (pH = 7.4; 0.1 M). For biofilm fixation, 100 µL of 99% methanol was added. The biofilm was then stained with 0.05% crystal violet for 15 min at room temperature. The excess was removed by two washes with ultra pure water. Purple coloration appeared after the addition of 100 μL of 33% acetic acid and was detected at 630 nm. The percentage of biofilm inhibition was determined by the equation below:

% inhibition = [100 − ((A_Sample_ − A_blank_)/(A_Control_ − A_blank_))] × 100 (1)

with A_Blank_ and A_Control_ corresponding to the absorbance of RPMI medium and untreated biofilm at 630 nm, respectively.

#### 4.4.4. Cytotoxicity Evaluation

Cytotoxicity EPS samples were tested on mammalian cell line Vero using MTT method according to Medjeldi et al. [67], measuring absorbance at 540 nm. The percentage of cytotoxic effect was calculated using the following equation:

[(A_Control_ − A_Sample_)/(A_Control_)] × 100. (2)

The 50% cytotoxic concentration (CC50), defined as the sample concentration able to reduce the cell viability by 50% when compared to an untreated control, was determined by linear regression analysis from a dose-response curve.

#### 4.4.5. Antiproliferative Effect of Different M_W_-EPS on Breast Cancer Cells

The exopolysaccharides samples were tested for their ability to inhibit the proliferation of the 4T1 murin breast carcinoma cells. The antiproliferative activity was determined by the XTT test as described in the Cell Proliferation Kit II (Sigma, 11465015001 Roche, St. Quentin Fallavier, France).

The results were expressed as a percentage of viability via the following formula:

% Viability = (A_test_/A_control_) × 100 (3)

A_Control_: Absorbance of untreated cells at 450 nm.

### 4.5. Statistical Analyses

Data were collected from three independent experiments assayed in triplicate and expressed as the mean ± standard deviation (SD). The statistical significance was analyzed by one-way ANOVA with STATISTICA. 5 software. The post-hoc test of Duncan multiple range was used to perform comparisons. Results were considered as statistically significant with a *p* < 0.05.

## 5. Conclusions

The present study demonstrated the biological activity of different molar masses exopolysaccharides from *Porphyridium marinum*. Interestingly, our findings clearly revealed the potentiality of native exopolysaccharide EPS-0C to inhibit the proliferation of Gram (+) and Gram (−) bacteria, as well as the formation of biofilm of *Candida albicans* at low concentration. However, the low molar masses exopolysaccharides EPS-2C and EPS-5C were found to be more effective for antiproliferative activity against breast cancer cells. Depending on the biological activity tested, this study also disclosed that these biological activities could be due to their molar masses, their viscosity as well as their composition (probably their content in sulfate and uronic acids). Thus, this study provides strong arguments to consider exopolysaccharides from *Porphyridium marinum* as a natural source of antibacterial, antibiofilm and anticancer products that are useful in pharmaceutical formulations and food industries as a natural preservative. Nevertheless, this study constitutes a first step to evaluate the potential of EPS from *P. marinum* in a drug development approach.

## Figures and Tables

**Table 1:** Macromolecular characterization of *P. marinum* EPS samples.

Samples	% Recovery	M_n_ (kDa)	M_w_ (kDa)	Rg (nm)	Rh (nm)	[ƞ] mL/g
EPS-0C	8.5	890 (±35%)	1400 (± 40%)	150 (± 5%)	67 (± 5.1)	1480 (± 1%)
EPS-2C	73	400 (± 0.7%)	550 (± 2%)	43 (± 2%)	26 (± 0.5)	230 (± 0.4%)
EPS-5C	72	340 (± 0.6%)	550 (± 4%)	41 (± 3%)	21 (± 0.6)	155 (± 0.5%)

M_w_: Weight-average molar mass estimated by SEC-MALLS-DRI. M_n_: Number-average molar mass estimated by SEC MALLS-DRI. Rg: Gyration radii. Rh: Hydrodynamic radii. [η]: Intrinsic viscosity.

**Table 2:** Uronic acids, sulfate content and monosaccharide composition of the different polysaccharidic samples.

	EPS Samples		
	EPS-0C	EPS-2C	EPS-5C
Uronic acids (%)	22 ± 0.1	22 ± 0.1	21 ± 0.2
Sulfates (%)	9.2 ± 0.3	8.7 ± 0.8	9.2 ± 0.7
Xylose (%)	47	47	44
Galactose (%)	25	25	29
Glucose (%)	20	19	20
Fucose (%)	1	1	1
Arabinose (%)	2	2	1
Glucuronic acid (%)	5	5	4

Uronic acids and sulfates content were determined by colorimetric assays and expressed as Eq.GlcA and Eq.SO_4_, respectively. Values are the average of at least 3 independent assays. Monosaccharide composition was obtained by HPAEC and results expressed as molar ratios.

**Table 3:** Antibacterial and anti-*Candida albicans* activities of *P. marinum* EPS samples.

Strains	MIC (µg/mL)				
	EPS-0C	EPS-2C	EPS-5C	Cefazolin	Amphotericin B
**Gram (−) Bacteria**					
*Escherichia coli* ATCC 25922	62.5	2500	2500	8	-
*Salmonella* Enteritidis	125	2500	-	4	-
**Gram (+) Bacteria**					
*Staphylococcus aureus* ATCC 29213	125	1250	2500	1	-
*Staphylococcus* Methicilin Resistant (SMR)	1000	-	-	512	-
**Yeast**					
*Candida albicans* ATCC 10231	-	-	-	-	0.125

MIC: Minimal Inhibitory Concentration. (-): Not determined. Cefazolin and Amphotericin B were used as positive controls.

## Data Availability

Data is contained within the article or Supplementary Materials.

## Supplementary Materials

The following are available online at https://www.mdpi.com/1660-3397/19/2/66/s1, Figure S1: HPAEC chromatograms of native (a: EPS-0C) and depolymerized EPS (b: EPS-2C, c: EPS-5C).
