# Supplementary material for: Potential of Exopolysaccharide from Porphyridium marinum to Contend with Bacterial Proliferation, Biofilm Formation, and Breast Cancer
Source: Mar Drugs. 2021 Jan 27;19(2):66. doi: 10.3390/md19020066 (PMC7911520; doi:10.3390/md19020066)
Supplement: Supplementary file 1 [file marinedrugs-19-00066-s001.pdf]

Supplementary Material

HPAEC chromatograms of native (a: EPS-0C) and depolymerized EPS (b: EPS-2C, c: EPS-5C)

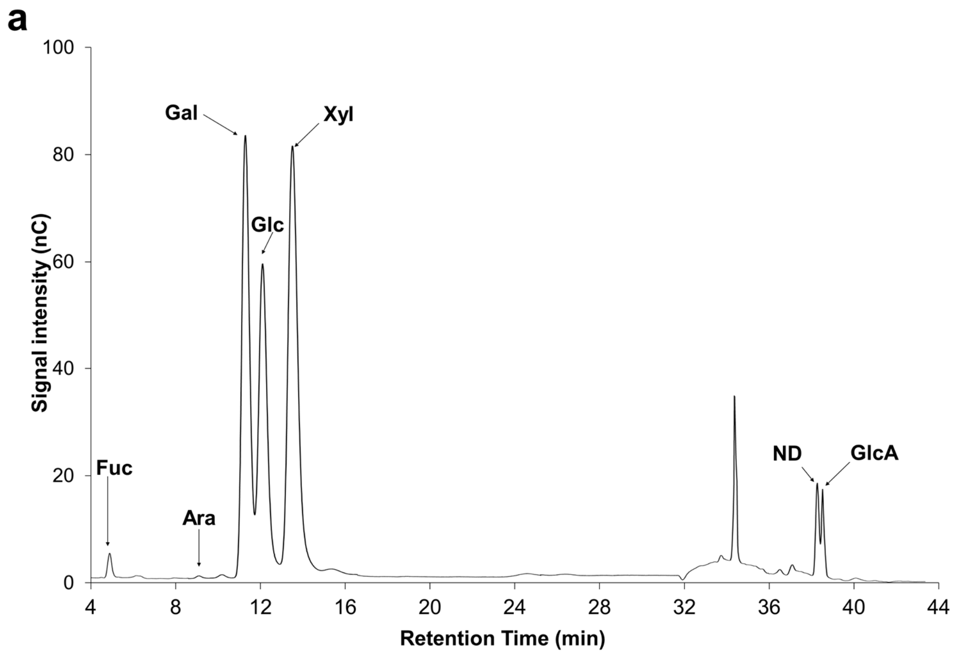

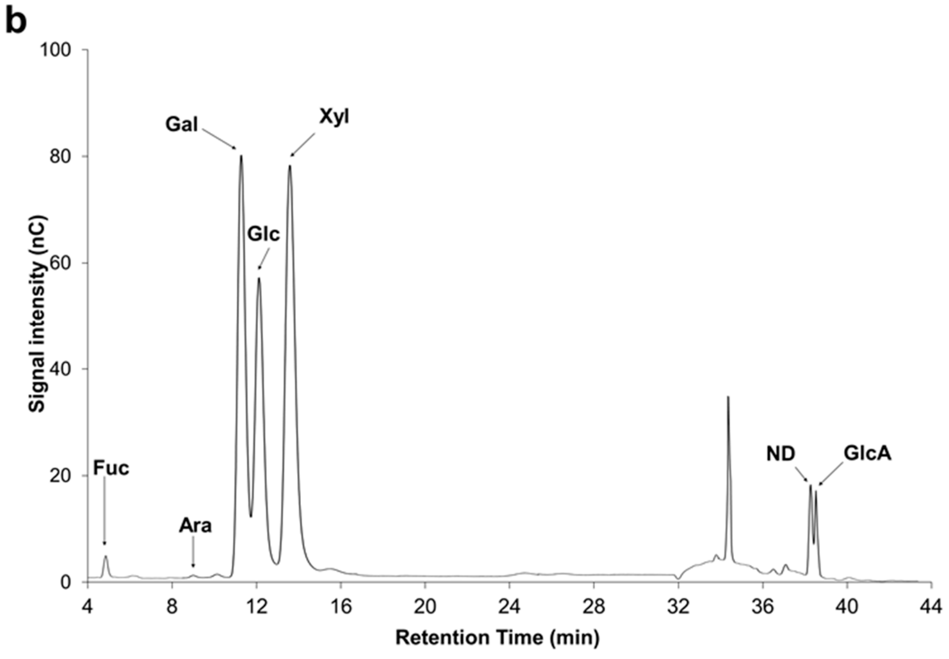

c

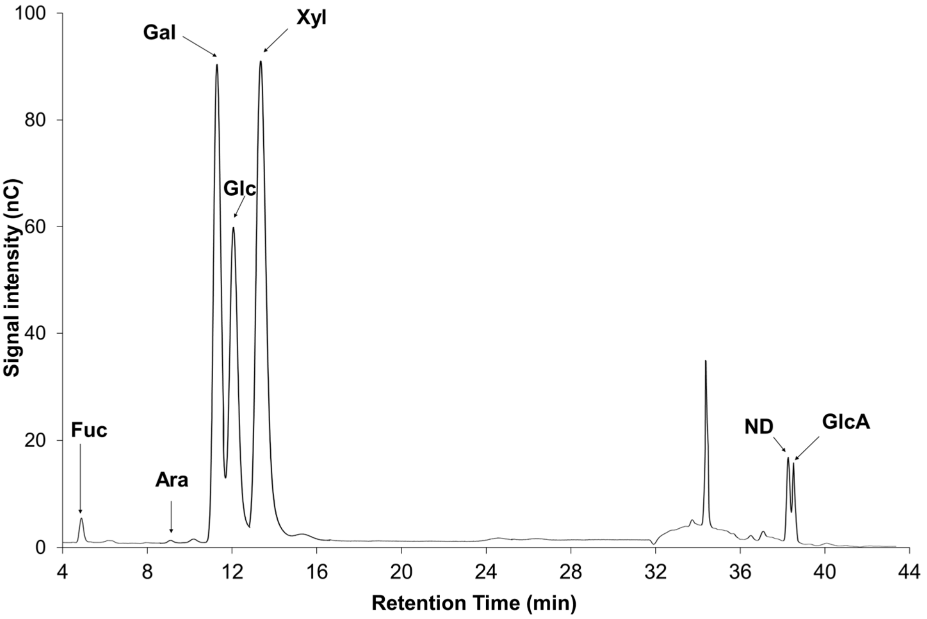
Gal
Xyl
Glc
Fuc
Ara
ND
GlcA
Signal intensity (nC)
Retention Time (min)
0
20
40
60
80
100
4
8
12
16
20
24
28
32
36
40
44
